# Selective Loss of Brain-Derived Neurotrophic Factor Exacerbates Brain Injury by Enhancing Neuroinflammation in Experimental *Streptococcus pneumoniae* Meningitis

**DOI:** 10.3389/fimmu.2020.01357

**Published:** 2020-06-26

**Authors:** Shengnan Zhao, Zhijie Zhang, Danfeng Xu, Yanfei Wang, Ling Li

**Affiliations:** Department of Pediatric Neurology, Xinhua Hospital Affiliated to Shanghai Jiaotong University School of Medicine, Shanghai, China

**Keywords:** *Streptococcus pneumoniae* meningitis, brain-derived neurotrophic factor, microglia/macrophage, neuroinflammation, brain injury

## Abstract

*Streptococcus pneumoniae* meningitis is a life-threatening bacterial infection of the central nervous system (CNS), and its unfavorable prognosis usually results from an intense inflammatory response. Recent studies have shown that brain-derived neurotrophic factor (BDNF) mediates anti-inflammatory and neuroprotective effects in CNS diseases; however, the distinct contribution of BDNF to pneumococcal meningitis (PM) remains unknown. In this study, we sought to investigate the effects of endogenous BDNF on the inflammatory response and brain damage in experimental PM. We used Camk2a-CreERT2 mice to delete *Bdnf* from the cerebral cortex and hippocampus, and meningitis was induced by intracisternal infection with *S. pneumoniae*. Clinical parameters were assessed during acute meningitis. At 24 h post-infection, histopathology, neutrophil granulocytes infiltration, and microglia/macrophage proliferation of brain tissues were evaluated. Additionally, cortical damage and hippocampal apoptosis were assessed using Nissl staining and terminal deoxynucleotidyl transferase dUTP-nick-end labeling (TUNEL), respectively. Pro-inflammatory cytokine levels were determined using real-time polymerase chain reaction (RT-PCR). Key molecules associated with the related signaling pathways were analyzed by RT-PCR and western blot. To investigate the role of microglia/macrophage in infected BDNF conditional knockout mice, GW2580 was used for microglia/macrophage depletion. Here, we, for the first time, found that BDNF conditional knockouts exhibited more profound clinical impairment, pathological severity, and neuron injury and enhanced microglia/macrophage proliferation than were observed in their littermate controls. Furthermore, the BDNF conditional knockouts showed an obviously increase in the expression of pro-inflammatory factors (*Tnf-*α, *Il-1*β, and *Il-6*). Mechanistically, loss of BDNF activated TLR2- and NOD2-mediated downstream nuclear factor kappa B (NF-κB) p65 and p38 mitogen-activated protein kinase (MAPK) pathways associated with *S. pneumoniae* infection. Furthermore, targeted depletion of microglia/macrophage population decreased the resistance of mice to PM with diminishing neuroinflammation in BDNF conditional knockouts. Our findings suggest that loss of BDNF may enhance the inflammatory response and contribute to brain injury during PM at least partially by modulating TLR2- and NOD2-mediated signaling pathways, thereby providing a potential therapeutic target for future interventions in bacterial meningitis pathologies.

## Introduction

Bacterial meningitis (BM) is a serious infection of the central nervous system (CNS) characterized by inflammation of the meninges and subarachnoid space (1). The most common causative pathogen of BM is *Streptococcus pneumoniae*, which accounts for nearly half of total cases (2). Even with conjugate vaccines and timely antibiotic therapy, pneumococcal meningitis (PM) has a high mortality rate ranging from 20 to 51% in the worldwide (3). About 50% survivors may present with brain damage and neurological deficits, such as sensory-motor deficits, hearing loss, aphasia, epilepsy, and cognitive impairment (4). These neurofunctional consequences during BM correlate with neuronal apoptosis, particularly in the hippocampus dentate gyrus (DG) (5). It has become evident that the development and progression of PM and the extensive tissue damage that occurs during infection are not simply due to the infectious agent itself but are also largely dependent on the intense activation of the host immune response (6). Bacterial lysis such as peptidoglycan, cell wall fragments generated during antibiotics treatment is highly immunogenic and may mediate neuronal cell injury and death by promoting the production of pro-inflammatory factors such as tumor necrosis factor (TNF)-α and interleukin (IL)−1β that exacerbate neurodegenerative pathology (7, 8). Possible therapeutic approaches to block TNF-α and/or IL-1β release are investigated in animal models of BM (9, 10).

Although the exact mechanism underlying immune activation in PM remains largely unclear, recent studies have indicated that pattern-recognition receptors (PRRs) can induce the release of inflammatory cascades that initiate brain immune damage (6, 11, 12). Toll-like receptor 2 (TLR2) recognizes various bacterial compounds and is identified as a main PRR involved in the detection of microbial infection (13). The activation of TLR2 results in the recruitment of the downstream signaling molecule myeloid differentiation factor 88 (MyD88), which subsequently upregulates the production of pro-inflammatory mediators (14). More importantly, TLR2 was involved in the pathogenesis of brain injury, and enhanced inflammation and associated hippocampal apoptosis were found after administration of a TLR2 agonist and pneumococci in wildtype mice but not in TLR2 knockout mice (15). We previously showed that MyD88-deficient mice display a markedly diminished inflammatory reaction in PM (16). In addition, pathogen-associated molecular patterns (PAMPs) are detected intracellularly by cytosolic receptors via nucleotide-binding oligomerization domain 2 (NOD2) (17). More importantly, emerging studies suggest the pivotal role of NOD2 activation in macrophage recruitment, phagocytosis, and *S. pneumoniae* clearance, while NOD2 deficiency results in reduced inflammatory reactions after *S. pneumoniae* infection *in vivo* and *in vitro* (18, 19). The activation of both TLR2 and NOD2 trigger the production of inflammatory cytokines, mainly including tumor TNF-α, IL-1β, and IL-6, via stimulation of the nuclear translocation of nuclear factor kappa B (NF-κB) as well as mitogen-activated protein kinases (MAPKs) (20, 21). Mounting evidence has shown that phosphorylated p38 MAPK and NF-κB p65 are involved in the *S. pneumoniae* induced expression of inflammatory mediators in the brain (22, 23). This overwhelming inflammatory response could cause severe injury to the brain, thus resulting in the frequently unfavorable prognosis reported during the progression of PM (24, 25). Hence, new adjunctive therapies capable of reducing inflammation and brain damage are needed to improve outcomes in PM.

Brain-derived neurotrophic factor (BDNF) is mainly distributed in the cerebral cortex and hippocampus and is a key member of the neurotrophin family that has demonstrated an ability to promote neural survival, development, differentiation, and plasticity in the CNS (26). There is increasing evidence from animal experiments and clinical studies indicating that BDNF exerts neuroprotective effects on bacterial meningitis. For example, in our previous study, we reported that the level of BDNF increased following administration of dexamethasone and further reduced mortality and sequelae in PM (27). Similarly, the serum and cerebrospinal fluid (CSF) BDNF levels were also increased in pediatric patients with bacterial meningitis (28). Furthermore, pretreatment with exogenous BDNF could improve the neurogenesis of endogenous neural stem cells (NSCs) in the hippocampus in a rat model of PM (29). Conversely, Barichello et al. (30) found that downregulating BDNF in the hippocampus following the long-term phase of meningitis was associated with behavioral deficits in experimental PM. In addition to its neuroprotective effects, we previously found that administration of exogenous BDNF suppressed the expression of inflammatory mediators after *S. pneumoniae* meningitis (31). A recent study demonstrated that BDNF prevented ischemia-induced apoptosis and inflammation *in vivo* by suppressing the TLRs/MyD88 signaling pathway (32). Therefore, BDNF has the double function in that it reduces both neuroinflammation and neurological sequelae and might constitute a promising adjuvant treatment for PM. We hypothesized that the loss of BDNF might contribute to an uncontrolled inflammatory response and subsequent brain damage. The early neonatal lethality of BDNF homozygous null knockouts mice prevents their use in further studies (33). To circumvent the neurodevelopmental problems associated with BDNF absence, we crossed floxed *Bdnf* mice with the Camk2a-driven CreERT2 strain to selectively delete BDNF in the cerebral cortex and hippocampus (34, 35). In this study, we investigate whether endogenous BDNF modulates localized inflammation and subsequent brain damage in the infected brain. We further clarify whether TLR2- and NOD2-mediated signaling pathways are involved in these processes. We report here that BDNF knockout results in the augmented activation of the TLR2 and NOD2 downstream molecules NF-κB p65 and p38 MAPK to significantly enhance the inflammatory response and brain injury in a murine model of PM.

## Materials and Methods

### Transgenic Mice and Experimental Design

Camk2a-CreERT2 mice express tamoxifen-inducible modified Cre recombinase in the neural tissues (including the cortex, hippocampus, and other structures). Homozygous floxed *Bdnf* mice (*Bdnf* ^F/F^; 021055, Jackson Laboratories) in a C57BL/6 background possess loxP sites flanking exon 5 in the *Bdnf* gene and are viable but obese and poor breeders. To produce forebrain-restricted BDNF mutant mice, *Bdnf*^*F*/+^ mice were interbred with Camk2a-CreERT2 mice to obtain *Bdnf*^*F*/+^; Camk2a-CreERT2 mice and these mice were mated to *Bdnf*^*F*/+^mice to obtain *Bdnf* conditional knockout mice (Camk2a-CreERT2/*Bdnf*^*F*/*F*^; Cre+/FF) as well as control mice (*Bdnf*^*F*/*F*^; Cre-/FF). Genotyping of mice for *Bdnf*^*F*/*F*^ and Camk2a-CreERT2 was performed by standard PCR using the following primers: Camk2a-CreERT2, (forward) 5′ACCTGGATGCTGACGAAGGC3′ and (reverse) 5′CGGTTATTCAACTTGCACCA3′; and Bdnf, (forward) 5′ GGTCTGAAATTACAAGCAGATGG3' and (reverse) 5′TGTCCGTGGACGTTTACTTCT3′. PCR products were loaded on agarose gels to check for the presence of the Camk2a-CreERT2 transgene (306 bp), the wild type *Bdnf* allele (198 bp), the *mutant* allele (~250 bp), and the heterozygote allele (198 bp and ~250 bp). Mice were injected intraperitoneally with the exogenous estrogen-receptor ligand tamoxifen (Sigma-Aldrich, St. Louis, MO, USA, catalog #T-5648) once per day for 5 consecutive days; then, mice were given a 2-week break before treatment sessions. The Cre-/FF littermate controls received the same treatment in parallel. The 6–8-week-old *Bdnf* conditional knockout mice and controls in a C57BL/6 background were used in our experiments. All experiments used both sex of mice at ~50:50 ratio. Before establishing the animal model of PM, 6 Cre+/FF mice and 6 Cre-/FF mice were used to determine BDNF expression in cortex, hippocampus, and brainstem. Animals were divided into the following groups (*n* = 8 per group): (1) Cre-/FF mice injected intracisternally with normal saline (NS); (2) Cre-/FF mice injected intracisternally with an *S. pneumoniae* suspension; (3) Cre+/FF mice injected intracisternally with NS; (4) Cre+/FF mice injected intracisternally with an *S. pneumoniae* suspension. The animals were housed at 23°C with 60% humidity under a 12 h/12 h light/dark cycle with food and tap water available *ad libitum*. The animal experiments were approved by the Animal Ethical and Welfare Committee of Xinhua Hospital affiliated with Shanghai Jiaotong University School of Medicine.

### Infecting Organisms

The standard serotype 3 strain *S. pneumoniae* (American Type Culture Collection, Manassas, VA, USA) was cultured on a sheep blood agar plate for 18 h followed by inoculation in VITAL AER broth overnight at 37°C in air with 5% CO_2_ to reach the logarithmic phase. The culture broth was centrifuged for 20 min at 5,000 g and resuspended twice in sterile saline to reach an approximate density of 1 × 10^4^ colony forming units (cfu)/ml using a nephelometer (Bio-Merieux, Marcy-l'Étoile, France).

### Mouse Model of *S. pneumoniae* Meningitis

The operation performed to induce *S. pneumoniae* meningitis has been previously described (16). Briefly, after all mice were anesthetized intraperitoneally with pentobarbital sodium (50 mg/kg), they were intracisternally injected with a 10 μl volume containing either 1 × 10^4^ cfu/ml viable *S. pneumoniae* or sterile saline. Briefly, animals were immobilized on a stereotaxic device under anesthesia. A natural indentation of cisterna magna can usually be touched on the midline of craniocervical junction where the microinjector is most likely to enter the occipital hole. At 24 h post-infection, the mice were weighed, and the severity of the disease was evaluated by assigning a clinical score in a blinded manner. For the clinical score, no apparent behavioral abnormality was scored as 0, moderate lethargy (an apparent decrease in spontaneous activity) as 1, severe lethargy (rare spontaneous movements, but walking after stimulation by the investigator) as 2, unable to walk as 3, and dead as 4 (16, 36). After clinical evaluation, the animals were anesthetized and perfused through the left ventricle with 50 ml of pyrogen-free saline. Bacterial titers were determined in samples of cerebellar homogenates by plating serial dilutions on the sheep blood agar plates under 37°C and 5% CO_2_ environments overnight. The brains were segment into two hemispheres. The left hemispheres were fixed in 4% paraformaldehyde overnight at 4°C for histological analysis. The brain tissues separated from the right hemispheres were frozen immediately and stored at −80°C for proteins and RNA preparation.

### *In vivo* Microglia/Macrophages Depletion

To evaluate the role of microglia/macrophages proliferation in the worsen outcomes caused by BDNF deficiency, GW2580 (Selleckchem, Houston, TX, USA, catalog #S8042), a selective inhibitor of the colony stimulating factor-1 (CSF-1) receptor kinase was used for microglia/macrophages depletion. GW2580 was dissolved in 0.5% hydroxypropyl methylcellulose and 0.1% Tween 80. Briefly, BDNF conditional knockout mice were divided into the following groups (*n* = 10 per group): (1) NS+0.5% hydroxypropyl methylcellulose/0.1% Tween 80; (2) PM+0.5% hydroxypropyl methylcellulose/0.1% Tween 80; (3) PM+GW2580; (4) NS+GW2580. PM was induced in BDNF conditional knockouts with 1 × 10^4^ cfu/ml *S. pneumoniae* as described above. Mice were treated with GW2580 at 80 mg/kg by oral gavage 1 day prior infection and every 12 h after *S. pneumoniae* infection until the animals were sacrificed 24 h later.

### Tissue Histopathological Evaluation

Paraffin-embedded brain tissues were sectioned coronally through the entire dentate gyrus using a microtome (4–6 μm thick). Brain inflammation and injury were analyzed by hematoxylin & eosin (H&E; Beyotime Biotechnology, Beijing, China, catalog #C0105), double-label immunofluorescence staining of Ly6G/IBA-1, and Nissl staining (Servicebio, Wuhan, China, catalog #G1036) according to the directions provided by the manufacturer. Terminal deoxynucleotidyl transferase dUTP nick end labeling (TUNEL) immunofluorescence staining was performed using an *in-situ* Cell Death Detection Kit (Roche, Basel, Switzerland, catalog #11684809910) to examine apoptosis-like cell death in the hippocampus DG. The numbers of TUNEL-stained nuclei and surviving neurons were counted blindly by two investigators.

### Immunohistochemical Staining

Immunohistochemical staining was performed to quantify BDNF, Ly6G and IBA-1 in the hippocampus and cerebral cortex. Briefly, brain sections were incubated with primary antibodies, including rabbit polyclonal anti-BDNF (1:800, Servicebio, Wuhan, China, catalog #GB11559), rabbit polyclonal anti-Ly6G (1:500, Servicebio, Wuhan, China, catalog #GB11229) or goat polyclonal anti-IBA-1 (1:500, Abcam, Cambridge, UK, catalog #ab5076) antibodies, overnight at 4°C. After three washes in phosphate-buffered saline (PBS) for 5 min each, the sections were incubated with secondary antibodies, including goat anti-rabbit IgG and donkey anti-goat IgG (Abcam, Cambridge, UK, catalog #ab150077, #ab150129), for 50 min at room temperature in a dark room and then washed twice with PBS. Finally, the sections were incubated with 4′,6-diamidino-2-phenylindole (DAPI; Beyotime Biotechnology, Beijing, China, catalog #C1002) for 10 min. Then, the sections were rinsed and visualized using a fluorescence microscope (Nikon, Japan).

### RNA Extraction and Real-Time Polymerase Chain Reaction (RT-PCR)

The brain tissues, including the hippocampus and cerebral cortex, were lysed, and RNA was extracted using a Total RNA Kit (TaKaRa, Shiga, Japan, catalog #9767) according to the manufacturer's protocol. The RNA was reverse-transcribed into cDNA using a PrimeScript One Step RT-PCR kit (TaKaRa, Shiga, Japan, catalog #RR036A). Subsequently, real-time PCR analyses were performed using a SYBR Premix Dimmer Eraser kit (TaKaRa, Shiga, Japan, catalog #RR420A) to assess cDNA template amplification on an ABI7500 system (Applied Biosystems, Carlsbad, CA, USA). The gene-specific primer sequences used in this study are listed in Table 1. The results for targeted gene expression in each sample were normalized to β-actin expression, and the 2^−ΔΔ*Ct*^ method was used to calculate the relative expression of messenger RNA (mRNA).

**Table 1:** The primers used in real-time PCR.

**Gene symbol**	**Forward**	**Reverse**	**Accession numbers**
Bdnf	TCATACTTCGGTTGCATGAAGG	AGACCTCTCGAACCTGCCC	NM_001048141
Ntf3	GGAGTTTGCCGGAAGACTCTC	GGGTGCTCTGGTAATTTTCCTTA	NM_001164035
Ntf5	TGAGCTGGCAGTATGCGAC	CAGCGCGTCTCGAAGAAGT	NM_198190
Tnf	CCCTCACACTCAGATCATCTTCT	GCTACGACGTGGGCTACAG	NM_013693
Il1b	GCAACTGTTCCTGAACTCAACT	ATCTTTTGGGGTCCGTCAACT	NM_008361
Il6	TAGTCCTTCCTACCCCAATTTCC	TTGGTCCTTAGCCACTCCTTC	NM_031168
Tlr2	GCAAACGCTGTTCTGCTCAG	AGGCGTCTCCCTCTATTGTATT	NM_011905
Myd88	TCATGTTCTCCATACCCTTGGT	AAACTGCGAGTGGGGTCAG	NM_010851
Nod2	CAGGTCTCCGAGAGGGTACTG	GCTACGGATGAGCCAAATGAAG	NM_145857
Actb	GGCTGTATTCCCCTCCATCG	CCAGTTGGTAACAATGCCATGT	NM_007393

### Western Blotting

Brain tissues (hippocampus and cortex) were sonicated (amplitude 38%, ultrasound 5 s, stop 3 s) in protein lysis buffer (Sigma-Aldrich, St. Louis, MO, USA, catalog #C3228) containing a Halt™ Protease Inhibitor Cocktail (Thermo Scientific, Waltham, MA, USA, catalog #87786) under ice water for five times and centrifuged at 12,000 g for 15 min at 4°C. The total protein concentration of each sample was determined using a BCA protein assay kit (Thermo Fisher Scientific, Waltham, MA, USA, catalog #23227) according to the manufacturer's instructions. Protein samples (50 μg) were resolved by 10% sodium dodecyl sulfate polyacrylamide (SDS-PAGE) gels and then transferred to polyvinylidene fluoride (PVDF) membranes (0.4 μm; Millipore, Billerica, MA, USA, catalog #IPVH00010). The membranes were blocked with 5% bovine serum albumin (BSA; Sangon Biotech, Shanghai, China, catalog #C500626) for 2 h at room temperature and probed at 4°C overnight with antibodies against TLR2 (1:1000; Abcam, Cambridge, UK, catalog #ab213676), NOD2 (1:500; Santa Cruz Biotechnology, Dallas, TX, catalog #sc-56168), MyD88, p38 MAPK, phospho-p38, NF-κB p65, phospho-p65 or β-actin (1:1000; Cell Signaling Technologies, Danvers, MA, USA, catalog #4283S, #8690, #4511, #8242, #3033, #3700). The membranes were rinsed with TBST for 3 × 15 min and incubated with secondary antibodies for 1 h at room temperature. Reactive signals were detected by ECL Western Blotting Substrate (Thermo Fisher Scientific, Waltham, MA, USA, #32109) and ChemiDoc™ XRS+ System (Bio-Rad, USA) and quantitated using Image Lab software version 3.0.

### Statistical Analysis

Differences between two groups were analyzed by two-tailed Student's *t-*tests. Multiple groups were compared using two-way analysis of variance (ANOVA; BDNF and meningitis) or one-way ANOVA followed by Tukey's *post-hoc* test. The data are expressed as the mean ± standard error of the mean (SEM). *p* < 0.05 was considered to indicate a significant difference. All graphs were generated using GraphPad Prism 5.0. Statistical analyses were performed using SPSS software version 17.0.

## Results

### Generation of Cortical and Hippocampal BDNF Knockout Mice

**Figure 1:**
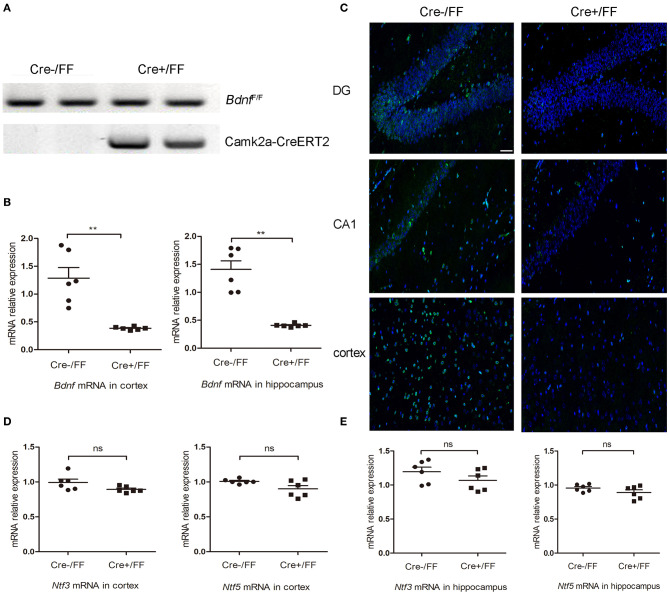
Selective deletion of BDNF in the cerebral cortex and hippocampus. **(A)** To produce forebrain-restricted BDNF mutant mice, *Bdnf*^*F*/+^ mice were interbred with Camk2a-CreERT2 mice. Standard PCR was used to detect the genotypes of Camk2a-CreERT2/*Bdnf*^*F*/*F*^ mice and *Bdnf*^*F*/*F*^ mice. Mice exhibited the floxed *Bdnf* band and the Camk2a-CreERT2 band were defined as Cre+/FF mice; mice only exhibited the floxed *Bdnf* band were defined as Cre-/FF littermate controls. **(B)** Mice were injected intraperitoneally with tamoxifen once per day for 5 consecutive days followed by a 2-week break. RT-PCR was used to compare the expression level of the *Bdnf* mRNA with that of actin in Cre+/FF mice and Cre-/FF littermate controls in the cortex and hippocampus (*n* = 6 mice per group). **(C)** Immunocytochemical staining for BDNF (green) and DAPI (blue) was performed in sections of the hippocampal CA1 and DG and the cortex areas in tissues obtained from Cre+/FF mice and Cre-/FF littermate control mice. The BDNF expression was reduced in the cortex and hippocampus in the Cre+/FF mice. **(D,E)** RT-PCR analysis of the expression of *Ntf3* and *Ntf5* mRNA in the cortex and hippocampus in mice with different genotypes. There were no differences between the genotypes in the expression levels of *Ntf3* and *Ntf5* in the cortex and hippocampus (*n* = 6 mice per group). ns: not significant (*p* > 0.05); **p < 0.01. Mean comparisons between groups were performed by two-tailed Student's *t-*tests. Error bars indicate SEM. The data shown are from three independent experiments. Scale bar: 50 μm.

To generate BDNF knockout mice, we crossed floxed *Bdnf* mice with Camk2a-CreERT2 mice. Subsequently, standard PCR was used to analyze the genotypes of offspring. A floxed *Bdnf* band and a Camk2a-CreERT2 band defined BDNF conditional knockouts (Cre+/FF). In contrast, the Cre-/FF littermate controls exhibited only a floxed *Bdnf* band (Figure 1A). BDNF levels were compared in control and conditional null mutants by RT-PCR and showed that the *Bdnf* mRNA was reduced by ~70 and 71% in the cortex and hippocampus, respectively, after tamoxifen injection (Figure 1B). In accordance with these findings, an immunofluorescence study showed that there were fewer BDNF-immunoreactive neurons in sections of the hippocampal DG, CA1 and cortex taken from Cre+/FF mice than in their Cre-/FF littermate controls (Figure 1C). To confirm that the deletion is very targeted, we measured BDNF expression in brainstem by RT-PCR and immunofluorescence staining. The results showed that there were no differences between the genotypes in the expression levels of BDNF in the brainstem (Figure S1). BDNF, neurotrophin (NT) −3 and NT-4/5 function through a common tropomyosin-receptor kinase B (TrkB) receptor (37, 38). To test whether the levels of these neurotrophic factors were increased to compensate for BDNF deletion, we measured the gene expression levels of these factors after tamoxifen administration. We found that there were no differences between the genotypes in the expression levels of *Ntf3* and *Ntf5* in the cortex (Figure 1D) and the hippocampus (Figure 1E).

### Conditional BDNF Deletion in Mice Aggravated the Clinical Severity of PM

As shown in Table 2, animals infected with *S. pneumoniae* showed positive bacterial cultures from cerebellar homogenates, whereas no pneumococci grew from cerebellar homogenates obtained from saline inoculated animals. Moreover, the infected Cre+/FF mice showed higher bacterial titers as compared with those from the infected Cre-/FF mice. Approximately 18 h after *S. pneumoniae* challenge, all infected mice began to display symptoms associated with PM, including weight loss, a lag in response speed and coma. Control animals injected with pyrogen-free saline showed a normal health status. Of the 8 infected animals in each PM group, one (1/8) animal died in the Cre-/FF group and two (2/8) animals died in the Cre+/FF group at 24 h (Table 2). Weight loss was significantly more pronounced in the Cre+/FF mice than in their Cre-/FF littermate controls (Table 2). In addition, clinical scores for disease severity showed markedly more severity in infected Cre+/FF mice than in control mice (Table 2).

**Table 2:** Bacterial titer, weight loss, clinical score, and mortality in different groups (mean ± standard error).

**Groups**	**Bacterial titer [log CFU/mL]**	**Weight loss (g)**	**Clinical score**	**Mortality**
Cre-/FF+NS	_	0.85 ± 0.22 (*n =* 8)	0	0/8
Cre-/FF+PM	4.18 ± 0.16 (*n =* 7)	3.51 ± 0.34^a^ (*n =* 7)	1.88 ± 0.35 (*n =* 8)	1/8
Cre+/FF+NS	_	1.83 ± 0.44 (*n =* 8)	0	0/8
Cre+/FF+PM	5.01 ± 0.06^c^ (*n =* 6)	5.48 ± 0.6^b^^,^ ^c^ (*n =* 6)	2.88 ± 0.30^d^ (*n =* 8)	2/8

Mice were intracisternally injected with a 10 μl volume containing either 10^4^ cfu/ml S. pneumoniae or sterile saline. Weight loss, clinical score, and mortality were evaluated at 24 h post-infection.

a p < 0.001, compared with Cre-/FF+NS.

b p < 0.001, compared with Cre+/FF+NS.

c p < 0.01, compared with Cre-/FF+PM.

d *p < 0.05, compared with Cre-/FF+PM*.

### Conditional BDNF Deletion in Mice Aggravated the Pathological Severity of PM

**Figure 2:**
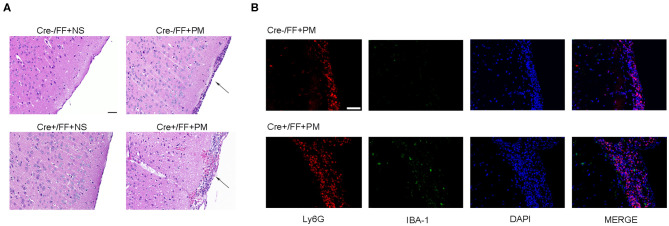
BDNF conditional knockout aggravated the pathological severity of PM. Mice were intracisternally injected with a 10 μl volume containing either 10^4^ cfu/ml *S. pneumoniae* or sterile saline. At 24 h post-infection, all survivals were sacrificed to harvest the brain. **(A)** H&E staining of coronal brain sections obtained in each group. Uninfected mice showed no inflammatory infiltrates, whereas all infected mice showed infiltration of inflammatory exudate in the subarachnoid space. Cre+/FF mice had a more aggravated pathological severity than was observed in their Cre-/FF littermate control mice. **(B)** Double-label immunofluorescence staining of Ly6G+ neutrophil granulocytes (red) and IBA-1+ microglia/macrophages (green). All infected mice showed massive infiltration of Ly6G+ neutrophil granulocytes into the subarachnoid space from the circulation in the acute phage of infection. Cre+/FF mice exhibited enhanced neutrophil granulocytes infiltration than were observed in their littermate controls. Scale bar: 50 μm.

When histopathology was examined at 24 h post-infection, large numbers of inflammatory cells were found to have infiltrated the subarachnoid space, and subarachnoidal and cortical hemorrhage was observed. Additionally, a significant increase in the number of cells infiltrated with inflammatory factors was observed in the brains of infected Cre+/FF mice than in their Cre-/FF littermate controls (Figure 2A). This finding was consistent with our previous study, which indicated that exogenous BDNF pretreatment alleviated pathological severity after pneumococcal infection (31). From the H&E stained slides, we observed that most exudative cells were infiltrated neutrophil granulocytes, which is a characteristic feature of acute bacterial meningitis. We performed double-label immunofluorescence staining of Ly6G+ neutrophil granulocytes and IBA-1+ microglia/macrophage and found massive infiltration of Ly6G+ neutrophil granulocytes into the subarachnoid space from the circulation. Moreover, BDNF conditional knockouts exhibited enhanced neutrophil granulocytes infiltration than were observed in their littermate controls at 24 h post-infection (Figure 2B).

### Conditional BDNF Deletion in Mice Increased Neuronal Injury and Hippocampal Apoptosis Associated With PM

**Figure 3:**
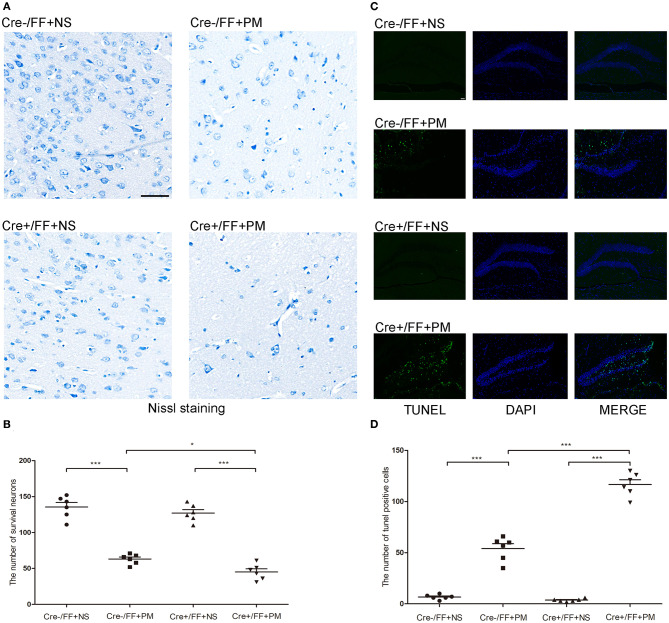
BDNF conditional knockout increased the neuronal injury and hippocampal apoptosis associated with PM. Mice were intracisternally injected with a 10 μl volume containing either 10^4^ cfu/ml *S. pneumoniae* or sterile saline. At 24 h post-infection, all survivals were sacrificed to harvest the brain. **(A)** The number of surviving neurons was assessed in the cerebral cortex by Nissl staining in all groups at 24 h post-infection. In infected Cre+/FF mice group, the neuron loss is more severe than in Cre-/FF mice. **(B)** Quantitative analysis of the Nissl results (*n* = 6 mice per group). **(C)** TUNEL staining was used to assess apoptotic neurons in the hippocampal DG. There are more TUNEL-positive cells in infected Cre+/FF mice than in infected control mice. **(D)** Quantitative analysis of the TUNEL results (*n* = 6 mice per group). *p < 0.05; ***p < 0.001. Mean comparisons between groups were performed by two-way ANOVA followed by Tukey's *post-hoc* test. Error bars indicate SEM. The data shown are from three independent experiments. Scale bar: 50 μm.

In recent years, studies have indicated that a wide spectrum of brain injuries are associated with PM, including focal necrosis of cortical neurons and apoptotic neuronal cell death in the DG, which are regularly detected in animals with PM (39). Therefore, we analyzed hippocampal apoptosis and neuronal injury in the cortex using TUNEL and Nissl staining, respectively. Our results indicated that cortical neuron loss was more remarkable in infected Cre+/FF mice than in control mice and that the knockouts also had more severely incomplete neuronal structures at 24 h post-infection (Figure 3A). There were significantly fewer surviving neurons in the infected Cre+/FF mice than in the Cre-/FF littermate controls (Figure 3B). Furthermore, *S. pneumoniae* infection caused obvious apoptosis in the hippocampal DG (Figure 3C), and the number of TUNEL-positive cells was significantly higher in infected Cre+/FF mice than in infected control mice (Figure 3D).

### Conditional BDNF Deletion in Mice Increased Microglia/macrophage Proliferation Associated With PM

**Figure 4:**
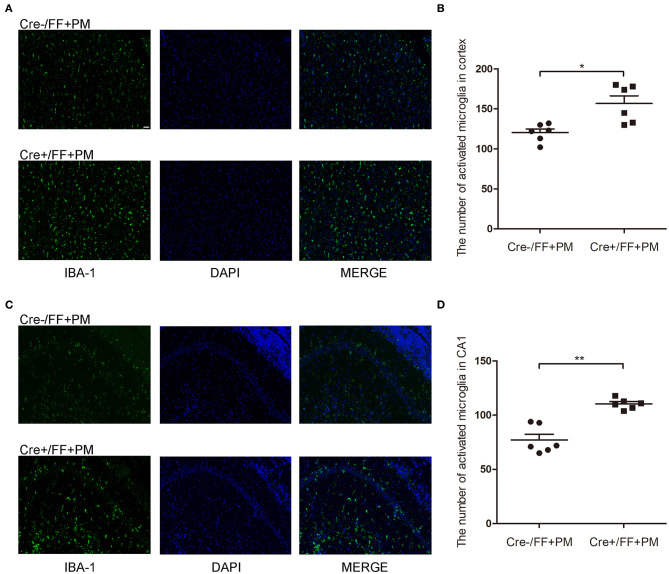
BDNF conditional knockout increased the microglia/macrophage proliferation associated with PM. Mice were intracisternally injected with a 10 μl volume containing either 10^4^ cfu/ml *S. pneumoniae* or sterile saline. At 24 h post-infection, all survivals were sacrificed to harvest the brain. **(A,C)** Immunofluorescent staining showing IBA-1 (green) positive cells counterstained with DAPI (blue) in the cortex and in the CA1 hippocampal region. All infected animals showed microglia/macrophage proliferation and the infected Cre+/FF mice showed significantly enhanced IBA-1 fluorescence intensity than was observed in the infected controls. **(B,D)** Quantitative analysis of IBA-1-positive cell numbers in the cortex and in the CA1 region of the hippocampus (*n* = 6 mice per group). *p < 0.05; **p < 0.01. Mean comparisons between groups were performed by two-tailed Student's *t-*tests. Error bars indicate SEM. The data shown are from three independent experiments. Scale bar: 50 μm.

IBA-1 staining was performed to evaluate the local status of inflammation in the CNS during PM. As shown in Figure 4, microglia/macrophage were detected in all infected animals after *S. pneumoniae* infection. Moreover, infected Cre+/FF mice showed significantly enhanced IBA-1 fluorescence intensity in the cortex (Figure 4A) and hippocampus CA1 (Figure 4C) than was observed in the infected controls, and the number of IBA-1 positive cells was significantly higher in infected Cre+/FF mice than in infected Cre-/FF littermate controls (Figures 4B,D).

### Conditional BDNF Deletion in Mice Upregulated Inflammatory Factor Expression Associated With PM in Brain Tissues

**Figure 5:**
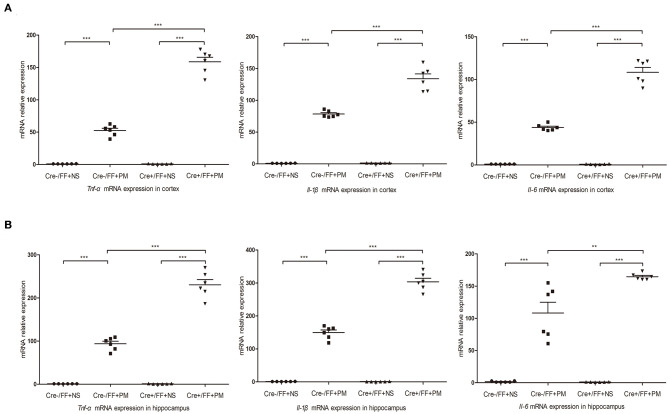
BDNF conditional knockout upregulated pro-inflammatory cytokine expression in PM. Mice were intracisternally injected with a 10 μl volume containing either 10^4^ cfu/ml *S. pneumoniae* or sterile saline. At 24 h post-infection, all survivals were sacrificed to harvest the brain. **(A,B)** The levels of pro-inflammatory cytokines (*Tnf-*α, *Il-1*β, and *Il-6*) were measured using RT-PCR in both the cortex and hippocampus at 24 h post-infection. In cortex and hippocampus homogenates from infected Cre+/FF mice, a significant increased expression of *Tnf-*α, *Il-1*β, and *Il-6* was detected than infected control mice (*n* = 6 mice per group). **p < 0.01; ***p < 0.001. Mean comparisons between groups were performed by two-way ANOVA followed by Tukey's *post-hoc* test. Error bars indicate SEM. The data shown are from three independent experiments.

At 24 h post-infection, brains (hippocampus and cerebral cortex) were harvested to examine the expression of proinflammatory factors (*Tnf-*α, *Il-1*β, and *Il-6*) by RT-PCR. The results of RT-PCR showed that the mRNA levels of all the analyzed inflammatory factors were significantly higher after infection. Moreover, the levels of inflammatory factors were also higher in Cre+/FF mice in the cortex (Figure 5A) and the hippocampus (Figure 5B) relative to the results observed in infected control mice. These data indicated that BDNF deletion was significantly associated with the expression of pro-inflammatory factors following exposure to pneumococcal infection.

### Conditional BDNF Deletion in Mice Upregulated TLR2 and NOD2 Expression Associated With PM

**Figure 6:**
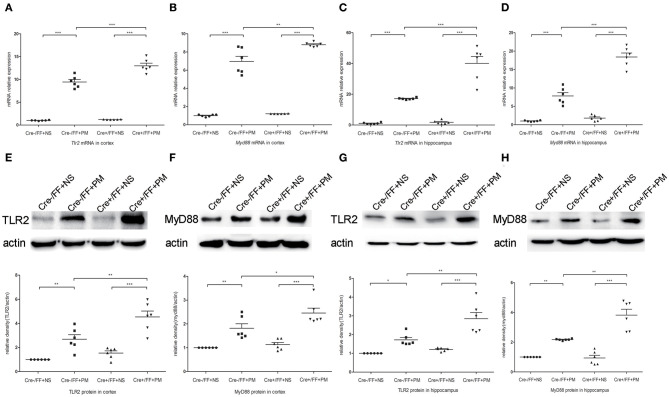
BDNF conditional knockout activated the TLR2 and MyD88 expression associated with PM. Mice were intracisternally injected with a 10 μl volume containing either 10^4^ cfu/ml *S. pneumoniae* or sterile saline. At 24 h post-infection, all survivals were sacrificed to harvest the brain. **(A–D)** The mRNA levels of *Tlr2* and *Myd88* mRNA were measured in the cortex and hippocampus by RT-PCR at 24 h post-infection (*n* = 6 mice per group). **(E–H)** The relevant protein expression in cortical and hippocampal tissue lysates and a density analysis of TLR2, MyD88, and β-actin expression in each group (*n* = 6 mice per group). BDNF conditional knockout increased levels of TLR2 and MyD88 in the cortex and hippocampus associated with PM. *p < 0.05; **p < 0.01; ***p < 0.001. Mean comparisons between groups were performed by two-way ANOVA followed by Tukey's *post-hoc* test. Error bars indicate SEM. The data shown are from three independent experiments.

**Figure 7:**
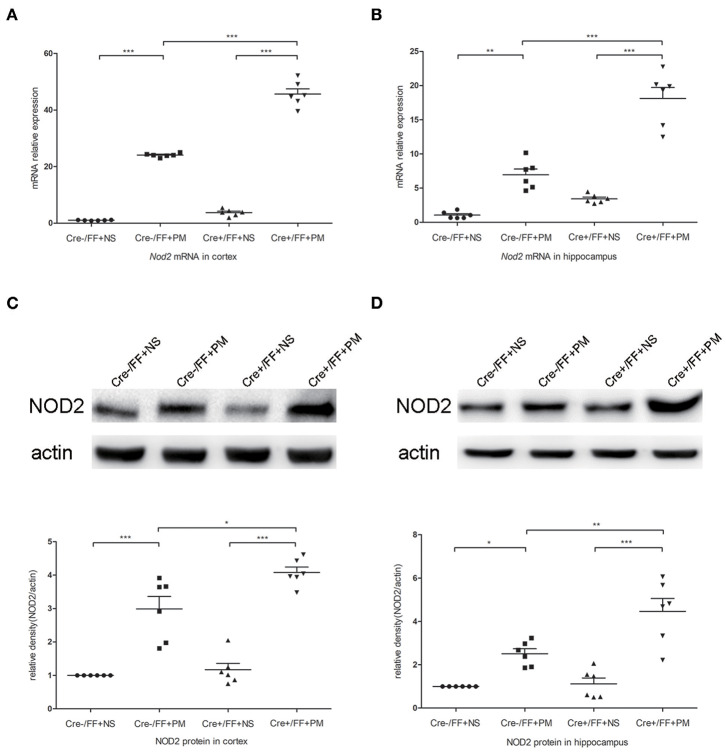
BDNF conditional knockout activated the NOD2 expression associated with PM. Mice were intracisternally injected with a 10 μl volume containing either 10^4^ cfu/ml *S. pneumoniae* or sterile saline. At 24 h post-infection, all survivals were sacrificed to harvest the brain. **(A,B)** The mRNA expression levels of *Nod2* in the cortex and hippocampus were assessed by RT-PCR at 24 h post-infection (*n* = 6 mice per group). **(C,D)** The relevant protein expression levels in cortical and hippocampal tissue lysates and a density analysis of NOD2 and β-actin expression were evaluated in each group (*n* = 6 mice per group). BDNF conditional knockout increased levels of NOD2 in the cortex and hippocampus associated with PM. *p < 0.05; **p < 0.01; ***p < 0.001. Mean comparisons between groups were performed by two-way ANOVA followed by Tukey's *post-hoc* test. Error bars indicate SEM. The data shown are from three independent experiments.

To investigate whether TLR2 and NOD2 are involved in the inflammatory response to *S. pneumoniae* infection in BDNF knockout mice, we measured the expression levels of NOD2 and TLR2 and the downstream molecules MyD88 in the brain tissues of mice challenged with *S. pneumoniae*. Compared with non-infected mice, mice submitted to *S. pneumoniae* stimulation expressed higher levels of *Tlr2* and *Myd88* mRNA at 24 h after infection in both the cortex (Figures 6A,B) and the hippocampus (Figures 6C,D). Additionally, the bacteria-induced upregulation of *Tlr2* and *Myd88* was markedly stronger in Cre+/FF mice than in control mice. Western blot and grayscale analyses were used to evaluate total TLR2 and MyD88 protein levels in the cortex (Figures 6E,F) and hippocampus (Figures 6G,H), and the results agreed with the findings for the mRNA levels of these markers as measured by RT-PCR. Similarly, there was a remarkable increase in the expression of the *Nod2* mRNA in response to pneumococcal stimulation in the cortex (Figure 7A) and hippocampus (Figure 7B), and Cre+/FF mice showed clearly higher expression levels of *Nod2* than were observed in control mice. Western blot and grayscale analyses were used to evaluate NOD2 protein levels in the cortex (Figure 7C) and hippocampus (Figure 7D), and the results agreed with the findings for the mRNA levels of this marker. These data indicated that TLR2 and NOD2 signaling are involved in the inflammatory response observed in Cre+/FF mice after pneumococcal infection.

### Conditional BDNF Deletion in Mice Further Activated NF-κB p65 and p38 MAPK in PM

**Figure 8:**
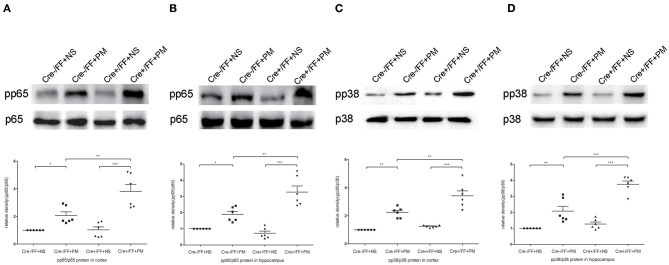
BDNF conditional knockout activated the NF-κB p65 and p38 MAPK signaling pathways in brain tissues. Mice were intracisternally injected with a 10 μl volume containing either 10^4^ cfu/ml *S. pneumoniae* or sterile saline. At 24 h post-infection, all survivals were sacrificed to harvest the brain. **(A–D)** The relevant protein expression of p65, pp65, p38, and pp38 in the hippocampus and cortex were evaluated in each group. The graphs indicated the expression ratios of pp65/p65 in the cortex and hippocampus as well as those of pp38/p38 (*n* = 6 mice per group). BDNF conditional knockout increased levels of phosphorylated p65 and p38 associated with PM. *p < 0.05; **p < 0.01; ***p < 0.001. Mean comparisons between groups were performed by two-way ANOVA followed by Tukey's *post-hoc* test. Error bars indicate SEM. The data shown are from three independent experiments.

We next investigated the influence of BDNF deletion on *S. pneumoniae*-induced activation of NF-κB p65 and p38 MAPK, which are two common downstream factors in both the TLR2 and the NOD2 signaling pathway (40, 41). Our data showed that *S. pneumoniae* infection activated the NF-κB pathway, as demonstrated by the substantially enhanced phosphorylation of p65 in the cortex (Figure 8A) and hippocampus (Figure 8B). The similar activation of the p38 MAPK pathway was also observed in all infected mice and was associated with the markedly enhanced phosphorylation of p38 in the cortex (Figure 8C) and hippocampus (Figure 8D). Moreover, the *S. pneumoniae*-induced phosphorylation of p65 and p38 was substantially stronger in the cortex and hippocampus of Cre+/FF mice exposed to *S. pneumoniae* infection than in their Cre-/FF littermate controls. These data indicated that BDNF deletion resulted in the augmented activation of both the NF-κB p65 and the p38 MAPK pathway in brain tissues during PM.

### Microglia/macrophage Depletion Attenuated Neuroinflammation but Aggravated Clinical Impairment in PM

**Figure 9:**
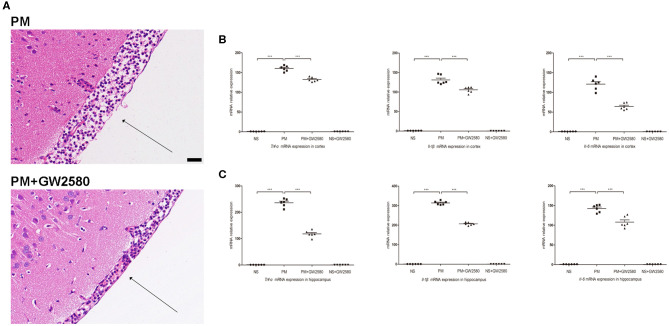
Macrophage/microglia depletion attenuated neuroinflammation in PM. BDNF conditional knockout mice were treated with GW2580 at 80 mg/kg by oral gavage 1 day prior infection and every 12 h after *S. pneumoniae* infection until the animals were sacrificed 24 h later. PM was induced with 10^4^ cfu/ml *S. pneumoniae* as described above. **(A)** H&E staining of coronal brain sections obtained in infected groups. All infected mice showed infiltration of inflammatory exudate in the subarachnoid space. PM+GW2580 mice had a decrease of leukocyte invasion in the subarachnoid space than was observed in PM control mice. **(B,C)** The levels of pro-inflammatory cytokines (*Tnf-*α, *Il-1*β, and *Il-6*) were measured using RT-PCR in both the cortex and hippocampus at 24 h post-infection. In cortex and hippocampus homogenates from PM+GW2580 mice, a significant decreased expression of *Tnf-*α, *Il-1*β, and *Il-6* was detected than PM control mice (*n* = 6 mice per group). ***p < 0.001. Mean comparisons between groups were performed by one-way ANOVA followed by Tukey's *post-hoc* test. Error bars indicate SEM. The data shown are from three independent experiments. Scale bar: 50 μm.

To assess the effect of GW2580 on the microglia/macrophage population in BDNF conditional knockout mice, we determined the expression of IBA-1 by immunofluorescence staining at 24 h post-infection. The increase in IBA-1-positive cell numbers after infection was significantly decreased with GW2580 treatment in cortex and hippocampus (Figure S2). The depletion of microglia/macrophages led to an increase of bacterial loads in the cerebellar homogenates and these higher bacterial titers resulted in more severe clinical symptoms and higher mortality (Table 3). However, we observed a decrease of leukocytes invasion in the subarachnoid space after *S. pneumoniae* infection in PM+GW2580 group compared with PM control group (Figure 9A). At 24 h post-infection, the mRNA expression of pro-inflammatory cytokines (i.e., *Tnf-*α, *Il-1*β, and *Il-6*) in both the cerebral cortex and hippocampus homogenates was evaluated by RT-PCR. Our results showed that *S. pneumoniae* infection led to massive pro-inflammatory cytokine release in BDNF conditional knockout mice. In contrast, the expression of *Tnf-*α, *Il-1*β, and *Il-6* was significantly attenuated in microglia/macrophage depletion mice, reflecting impaired immune activation (Figures 9B,C).

**Table 3:** Bacterial titer, weight loss, clinical score, and mortality in different groups (mean ± standard error).

**Groups**	**Bacterial titer** **[log CFU/mL]**	**Weight loss (g)**	**Clinical score**	**Mortality**
NS	_	0.78 ± 0.25 (*n =* 10)	0	0/10
PM	5.05 ± 0.22 (*n =* 8)	5.14 ± 0.57^a^ (*n =* 8)	2.7 ± 0.24 (*n =* 10)	2/10
PM + GW2580	5.64 ± 0.15^c^ (*n =* 6)	7.15 ± 0.94^b^ (*n =* 6)	3.4 ± 0.16^b^ (*n =* 10)	4/10
NS + GW2580	_	0.41 ± 0.16 (*n =* 10)	0	0/10

BDNF conditional knockout mice were treated with GW2580 at 80 mg/kg by oral gavage 1day prior infection and every 12 h after S. pneumoniae infection until the animals were sacrificed 24 h later. PM was induced with 10^4^ cfu/ml S. pneumoniae as described above. Weight loss, clinical score, and mortality were evaluated at 24 h post-infection.

a p < 0.001, compared with NS.

b p < 0.05, compared with PM.

c *p < 0.001, compared with PM*.

## Discussion

In this study, we conditionally deleted the *Bdnf* gene from the cortex and hippocampus in mice to determine the contribution of this marker to *S. pneumoniae* infection processing. The results of this study demonstrated that conditional BDNF knockout aggravated the clinical and pathological severity observed after *S. pneumoniae* infection and intensified the inflammatory response, neuron injury and hippocampal apoptosis that are correlated with *S. pneumoniae* meningitis. Additionally, we found that BDNF deletion enhanced neurological impairment at least partially by modulating the TLR2 and NOD2 signaling pathways. Targeted depletion of microglia/macrophage population attenuated neuroinflammation during PM, however, it failed to reverse the poor outcomes of infected BDNF conditional knockout mice. Collectively, our results suggested a novel regulatory mechanism by which the inflammatory response was mediated by endogenous BDNF in PM, and these results increased our understanding of the pathogenesis of this disease.

Tissue-specific gene knockout can yield remarkable information about the relative therapeutic targets in a disease, and time-specific gene ablation surmounts potential physiological compensatory mechanisms that can cover the true phenotypic contribution of a gene (42, 43). Camk2a is a calcium-activated serine/threonine kinase that is abundantly present in the forebrain (including the cortex, hippocampus, and other structures), but is mostly absent from mid-hind brain regions. It is widely used to drive a tamoxifen-inducible CreERT2 recombinase construct that permits tissue-specific gene deletion in adult animals (44, 45). BDNF has a wide array of functions in the brain and is expressed at the highest levels in the hippocampus and cerebral cortex (46, 47). Here, to identify the role of endogenous BDNF in the inflammatory reaction and brain injury during PM, we crossed floxed *Bdnf* mice with the Camk2a-driven CreERT2 strain to delete *Bdnf* . RT-PCR was used to assess the effects of conditional *Bdnf* deletion, and the results showed that tamoxifen injection reduced the mRNA expression level of *Bdnf* by ~70% in the cortex and hippocampus. The remaining ~30% of Bdnf mRNA may be produced by cells that did not undergo gene recombination (34). Although BDNF, NT-3 and NT-4/5 all activate the TrkB receptor, there were no differences in the expression levels of *Ntf3* or *Ntf5* between Cre+/FF mice and their Cre-/FF littermate controls. These results showed that *Ntf3* and *Ntf5* expression levels were not upregulated to compensate for the absence of BDNF after *Bdnf* gene deletion, in line with previous findings (34).

Despite the fact that significant progress has been made toward treatments for PM, the mortality and neurological sequelae rates associated with PM remain high (48, 49). The brain damage caused by *S. pneumoniae* infection is correlated with an enhanced inflammatory response in the CNS, especially after antibiotic treatment, which results in large amounts of bacterial components being released and recognized by brain-resident immune cells (14, 50). Subsequently, bacterial lysis stimulates the release of pro-inflammatory cytokines, such as TNF-α and IL-1β in the CNS, eventually resulting in mitochondrial dysfunction, neuronal apoptosis, and brain damage (51). In recent decades, therapeutic targets have popularly targeted the regulation of the inflammatory response and eliminating side effects correlated with the excessive activation of the immune system. Although numerous therapeutic adjunctive approaches have been investigated to decrease the harmful effects of the inflammatory response *in vitro* and in animal models treated with corticosteroids, IL-1β receptor antagonists, or TNF-α inhibitors, the clinical efficacy of these treatments remains unsatisfactory (24, 49, 52). Recent studies have indicated that BDNF exerts neuroprotective effects in diverse CNS-related diseases, and this may provide a promising adjunctive therapy for acute BM. Our recent study verified that BDNF administration decreased the inflammatory response and hippocampal apoptosis by modulating immune-related intracellular signaling pathways in rats submitted to PM (31). In addition, we demonstrated that BDNF was obviously upregulated after *S. pneumoniae* inoculation for 24 h but then declined with time (53). Moreover, the expression of BDNF in brains was further reduced after infected animals were treated with antibiotics but was increased when the animals were treated with an antibiotic plus dexamethasone (53, 54). These findings suggested that decreased BDNF levels were associated with the aggravation of infection-induced intracranial complications; however, direct evidence for the involvement of endogenous BDNF in *S. pneumoniae* meningitis is lacking.

In our animal model, the intracisternal injection with 10^4^ cfu/ml of standard serotype 3 *S. pneumoniae* induced pathological manifestations that resemble the human situation: microglial activation, neutrophil granulocytes recruitment, and indicated neuronal injury in the cortex and hippocampus (55). Although, the unphysiological mode of bacterial infection is a theoretical disadvantage, the directly injection of bacteria into CSF is easy to perform and ensures all bacterial fluid distributed to the intracranial compartments. In contrast, the bacteremia-derived meningitis model may result in rapid bacterial clearance from the bloodstream not necessarily leading to BM (data not shown).

In the present study, we showed that the loss of BDNF decreased the resistance of mice to *S. pneumoniae* meningitis and was associated with more weight loss and worse clinical features. There was a tendency for infected Cre+/FF mice to have a lower survival rate than their littermate controls. We suggest that more animals and the complete removal of the *Bdnf* gene will be required to further analyze this difference in mortality.

BDNF deletion resulted in increased microglia/macrophage proliferation and neutrophil infiltration of subarachnoid space during *S. pneumoniae* infection than was observed in infected control mice. Activated microglia/macrophage can produce large amounts of cytokines and neurotoxic mediators, leading to neuronal cell injury and even the perpetuation of neuronal damage (56). These results are in accordance with the finding that overwhelmingly higher amounts of pro-inflammatory cytokines (TNF-α, IL-1β, and IL-6) are produced and released by immune cells in Cre+/FF mice than in their infected Cre-/FF littermate controls. Microglia activation is widely regarded as a double-edged sword possessing both protective and destructive effects. In our present study, targeted depletion of microglia/macrophage cell population decreased the resistance of mice to PM with diminishing neuroinflammation in BDNF conditional knockout mice. Once sensing the invasion of *S. pneumoniae*, microglia transform into activated phenotypes, and proliferate at the site of injury (57). The activated microglial cells release cytokines or chemokines that induce the infiltration of circulating immune cells during infection. Infiltrated leukocytes support bacterial clearance but also contribute to neuronal damage (58). The severity of clinical symptoms during PM depends on both the bacterial load and the host immune response. In the BDNF conditional knockout mice, the overgrowth of bacterial caused by microglia/macrophage depletion may be more detrimental for the outcomes of PM than the dismissed host immune response. Although our study found that BDND deficiency led to enhanced microglia/macrophage proliferation after infection, it may also result in other effects (e.g., increased bacterial tiers, neutrophil infiltration, activation of inflammatory pathway, and reduced neuroprotective effect) that contributes to the worsen outcomes in our animal model.

We found that the number of surviving neurons in the cortex was significantly lower and the number of apoptotic bodies was higher in the DG of infected Cre+/FF mice than in infected control mice. Significant brain damage arises from the mechanisms involved in neuronal apoptosis, especially in the hippocampus during PM, and autopsies performed in cases of PM have revealed that neuronal apoptosis occurs in the DG in this disease (5). Although a growing body of evidence has shown that BDNF participates in regulating intracellular signaling molecules to inhibit the expression of pro-inflammatory cytokines during infection, the exact mechanism by which BNDF exerts its effects in *S. pneumoniae* infection remains unclear (59).

**Figure 10:**
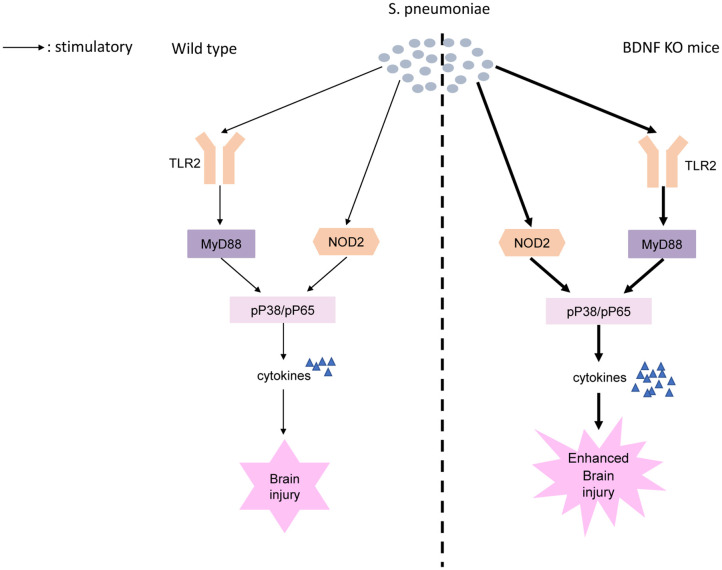
Proposed model of BDNF knockout exacerbates neuroinflammation and brain injury in experimental pneumococcal meningitis. *S. pneumoniae* is recognized by PRRs including TLR2 and NOD2. BDNF deletion results in a strong increase in the *S. pneumoniae*-induced phosphorylation of NF-κB p65 and phosphorylation of p38 MAPK through TLR2/MyD88 and NOD2 downstream signaling. Increased phosphorylation of NF-κB p65 and phosphorylation of p38 MAPK, as two of the major factors responsible for the transcription of the targeted inflammatory genes, lead to the excessive secretion of pro-inflammatory cytokines, thereby exacerbating brain inflammation and further injury.

We found BDNF deletion resulted in significantly higher post-*S. pneumoniae* infection levels of TLR2/MyD88 and NOD2 expression than were observed in infected control mice. TLR2 and NOD2 act as critical PRRs by detecting bacterial molecules. Upon activation of these receptors, they induce the activation of NF-κB and MAPK pathways, which eventually results in the transcription of the targeted inflammatory genes and production of proinflammatory cytokines (39, 60). Previous research has shown that *in vivo*, the selective activation of TLR2 led to inflammatory changes and high leukocyte concentrations in the CNS and neuronal apoptosis in the hippocampal DG. Of note, the TLR2-deficient mice submitted to *S. pneumoniae* showed lower levels of CSF pleocytosis and less excess neuronal damage than were found in controls (15). Similarly, MyD88-decient mice showed an obviously compromised inflammatory response, including reduced leucocyte infiltration and lower levels of cytokine release during *S. pneumoniae* infection (61). Moreover, the increase in NOD2 expression observed after *S. pneumoniae* infection further promotes inflammatory cell infiltration and blood-brain barrier (BBB) permeability within the CNS, leading to cerebral injury (12, 62). In the present study, we further demonstrated that BDNF deletion resulted in a strong increase in the *S. pneumoniae*-induced phosphorylation of NF-κB p65 and the phosphorylation of p38 MAPK than were observed in control mice. NF-κB p65 is a widely expressed transcription factor that is a strong inducer of many proinflammatory genes that are associated with the pathogenesis of PM. Early studies revealed that in PM, the pharmacological inhibition of NF-κB p65 obviously reduced the host inflammatory response and attenuated CNS complications, such as BBB disruption and cerebrovascular failure (63). Moreover, increased p38 MAPK activation was observed after stimulation with pneumolysin, and the inhibition of p38 resulted in a strong improvement in cell survival (64). Importantly, p38 MAPK is a critical regulator of inflammatory cytokine production, and some p38 MAPK-blocking drugs that reduce IL-1 and TNF-α expression have been successfully used in the treatment of inflammatory diseases (65, 66). Here, we provide the first evidence showing that BDNF deletion resulted in a strong increase in the *S. pneumoniae*-induced phosphorylation of NF-κB p65 and phosphorylation of p38 MAPK through TLR2/MyD88 and NOD2 downstream signaling, lead to the excessive secretion of pro-inflammatory cytokines, thereby exacerbating brain inflammation and further injury (Figure 10).

The role of BDNF may change over time as the infection evolves. There is also strong evidence to suggest important roles of BDNF in regulating neurogenesis, survival, plasticity, and neuronal differentiation (67, 68). Our previous studies showed that BDNF adjunctive treatment with antibiotics for 7 d could rescue cortical and hippocampal neurons in a rat model of pneumococcal meningitis (69). Moreover, administration of exogenous BDNF for 7–28 days might increase the proliferation and differentiation of NSCs in the hippocampus after bacterial meningitis (29). Our current study only evaluated the acute phase of the disease, and in subsequent studies, the role of endogenous BDNF at different time points during infection process should be investigated.

## Conclusion

In summary, the results of our study indicate that conditional *Bdnf* deletion exacerbates brain damage and augments the innate immunity-associated inflammatory response, likely by activating the TLR2- and NOD2-mediated downstream signaling pathways during *Streptococcus pneumoniae* meningitis; therefore, BDNF may be a promising candidate therapeutic target in BM.

## Data Availability Statement

The datasets generated for this study are available on request to the corresponding author.

## Ethics Statement

The animal experiments were approved by the Animal Ethical and Welfare Committee of Xinhua Hospital (approval ID: 2014041).

## Author Contributions

SZ performed research, analyzed data, and wrote the manuscript. ZZ and DX helped with the animal experiment. YW helped with the molecular biological experiments. LL provided the necessary guidance on the performance of all the experiment. All authors contributed to the article and approved the submitted version.

## Conflict of Interest

The authors declare that the research was conducted in the absence of any commercial or financial relationships that could be construed as a potential conflict of interest.

## Abbreviations

CNS: Central nervous system
BDNF: Brain-derived neurotrophic factor
PM: Pneumococcal meningitis
BM: Bacterial meningitis
PRRs: Pattern-recognition receptors
TLR2: Toll-like receptor 2
MyD88: Molecule myeloid differentiation factor 88
NOD2: Nucleotide-binding oligomerization domain 2
TNF: Tumor necrosis factor
IL: Interleukin
NF-κB: Nuclear factor kappa B
MAPKs: Mitogen-activated protein kinases
NSCs: Endogenous neural stem cells
NS: Normal saline
TUNEL: terminal deoxynucleotidyl transferase dUTP-nick-end labeling
DG: Dentate gyrus
NT-3: Neurotrophin-3
NT-4: Neurotrophin-4
TrkB: Tropomyosin-receptor kinase B
BBB: Blood-brain barrier
PAMPs: Pathogen-associated molecular patterns.
