# Supplementary material for: Selective Loss of Brain-Derived Neurotrophic Factor Exacerbates Brain Injury by Enhancing Neuroinflammation in Experimental Streptococcus pneumoniae Meningitis
Source: Front Immunol. 2020 Jun 26;11:1357. doi: 10.3389/fimmu.2020.01357 (PMC7333737; doi:10.3389/fimmu.2020.01357)
Supplement: Supplementary file 1 [file Data_Sheet_1.PDF]

Supplementary Material

1 Supplementary Figures and Tables

1.1 Supplementary Figures

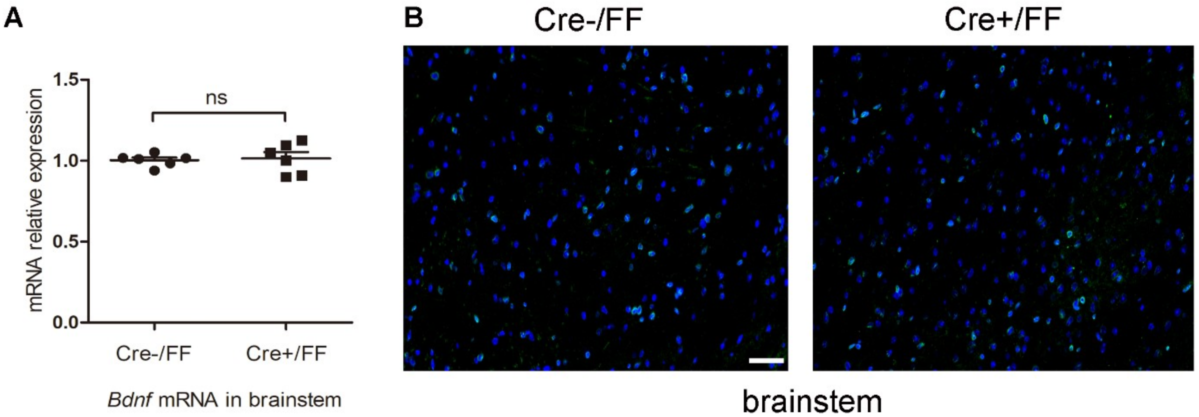

Supplementary Figure 1. BDNF expression in brainstem showed no differences between the genotypes. (A) Mice were injected intraperitoneally with tamoxifen once per day for five consecutive days followed by a two-week break. RT-PCR was used to compare the expression level of the Bdnf mRNA with that of actin in Cre+/FF mice and Cre-/FF littermate controls in the brainstem (n = 6 mice per group). (B) Immunocytochemical staining for BDNF (green) and DAPI (blue) was performed in sections of the brainstem in tissues obtained from Cre+/FF mice and Cre-/FF littermate control mice. There were no differences between the genotypes in the expression levels of BDNF in the brainstem (n = 6 mice per group). ns: not significant ($p > 0.05$). Mean comparisons between groups were performed by two-tailed Student's t-tests. Error bars indicate SEM. The data shown are from three independent experiments. Scale bar: 50 μm.

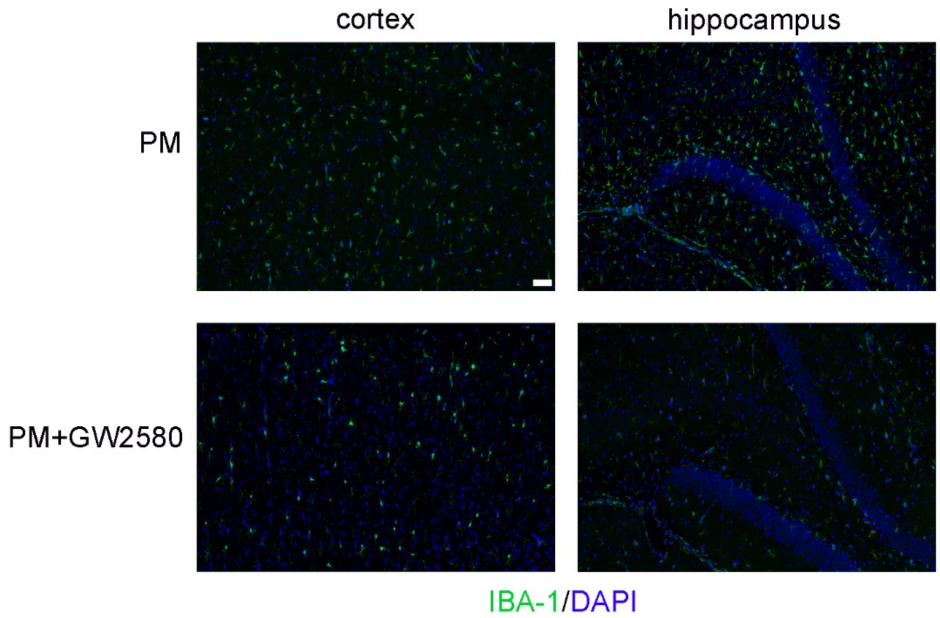

Supplementary Figure 2. GW2580 effectively depleted microglia/macrophage population in infected BDNF conditional knockout mice. BDNF conditional knockout mice were treated with GW2580 at 80 mg/kg by oral gavage one day prior infection and every 12 hours after *S. pneumoniae* infection until the animals were sacrificed 24 h later. PM was induced with 10^4 cfu/ml *S. pneumoniae* as described above. Paraffin sections of brain tissues were stained by immunofluorescence for microglia/macrophage (IBA-1) in the cortex and hippocampus. The increase in IBA-1-positive cell (green) numbers after infection was significantly decreased with GW2580 treatment in cortex and hippocampus. Scale bar: 50 µm.
